# Supplementary material for: Comparative multiomics analysis of cell physiological state after culture in a basket bioreactor
Source: Sci Rep. 2022 Nov 23;12:20161. doi: 10.1038/s41598-022-24687-4 (PMC9686226; doi:10.1038/s41598-022-24687-4)
Supplement: Supplementary file 1 — Supplementary Information 1. [file 41598_2022_24687_MOESM1_ESM.zip › raw data/Proteomic raw data/6.KEGGenrichment/EG.vs.CG/UP/pathway/src/00600.html]

map00600


- K00654, 2.3.1.50, R01281"

- Up regulated proteins


A0A0D9RHA9(Description:Aminotran\_1\_2 domain-containing protein;Gene:SPTLC3;log2FC:inf)

- K04628, 2.4.1.47, R01500"

- Up regulated proteins

A0A0D9QUW7(Description:Glucuronosyltransferase;Gene:UGT8;log2FC:1.1559)

Close
